# Prognostic Factors and a New Prognostic Index Model for Children and Adolescents with Hodgkin’s Lymphoma Who Underwent Autologous Hematopoietic Stem Cell Transplantation: A Multicenter Study of the Turkish Pediatric Bone Marrow Transplantation Study Group

**DOI:** 10.4274/tjh.2015.0280

**Published:** 2016-12-01

**Authors:** Vural Kesik, Erman Ataş, Musa Karakükcü, Serap Aksoylar, Fatih Erbey, Nurdan Taçyıldız, Alphan Küpesiz, Haldun Öniz, Ekrem Ünal, Savaş Kansoy, Gülyüz Öztürk, Murat Elli, Zühre Kaya, Emel Ünal, Volkan Hazar, Şebnem Yılmaz Bengoa, Gülsün Karasu, Didem Atay, Ayhan Dağdemir, Hale Ören, Ülker Koçak, M. Akif Yeşilipek

**Affiliations:** 1 Gülhane Training and Research Hospital Clinic of Pediatric Oncology, Ankara, Turkey; 2 Erciyes University Faculty of Medicine, Department of Pediatric Hematology-Oncology and Bone Marrow Transplantation Unit, Kayseri, Turkey; 3 Ege University Faculty of Medicine, Department of Pediatric Oncology and Bone Marrow Transplantation Unit, İzmir, Turkey; 4 Medical Park Bahçelievler Hospital, Pediatric Bone Marrow Transplantation Unit, İstanbul, Turkey; 5 Ankara University Faculty of Medicine, Department of Pediatric Oncology and Bone Marrow Transplantation Unit, Ankara, Turkey; 6 Akdeniz University Faculty of Medicine, Department of Pediatric Hematology-Oncology and Bone Marrow Transplantation Unit, Antalya, Turkey; 7 Tepecik Training and Research Hospital, Clinic of Pediatric Oncology and Bone Marrow Transplantation Unit, İzmir, Turkey; 8 Ondokuz Mayıs University Faculty of Medicine, Department of Pediatric Oncology and Bone Marrow Transplantation Unit, Samsun, Turkey; 9 Gazi University Faculty of Medicine, Department of Pediatric Hematology and Bone Marrow Transplantation Unit, Ankara, Turkey; 10 Dokuz Eylül University Faculty of Medicine, Department of Pediatric Hematology and Bone Marrow Transplantation Unit, İzmir, Turkey; 11 Medical Park Göztepe Hospital, Pediatric Bone Marrow Transplantation Unit, İstanbul, Turkey

**Keywords:** Childhood Hodgkin’s lymphoma, prognosis, Autologous hematopoietic stem cell transplantation, Prognostic index

## Abstract

**Objective:**

The prognostic factors and a new childhood prognostic index after autologous hematopoietic stem cell transplantation (AHSCT) in patients with relapsed/refractory Hodgkin’s lymphoma (HL) were evaluated.

**Materials and Methods:**

The prognostic factors of 61 patients who underwent AHSCT between January 1990 and December 2014 were evaluated. In addition, the Age-Adjusted International Prognostic Index and the Childhood International Prognostic Index (CIPI) were evaluated for their impact on prognosis.

**Results:**

The median age of the 61 patients was 14.8 years (minimum-maximum: 5-20 years) at the time of AHSCT. There were single relapses in 28 patients, ≥2 relapses in eight patients, and refractory disease in 25 patients. The chemosensitivity/chemorefractory ratio was 36/25. No pretransplant radiotherapy, no remission at the time of transplantation, posttransplant white blood cell count over 10x10^3^/µL, posttransplant positron emission tomography positivity at day 100, and serum albumin of <2.5 g/dL at diagnosis were correlated with progression-free survival. No remission at the time of transplantation, bone marrow positivity at diagnosis, and relapse after AHSCT were significant parameters for overall survival.

**Conclusion:**

The major factors affecting the progression-free and overall survival were clearly demonstrated. A CIPI that uses a lactate dehydrogenase level of 500 IU/L worked well for estimating the prognosis. We recommend AHSCT at first complete remission for relapsed cases, and it should also be taken into consideration for patients with high prognostic scores at diagnosis.

## Abstract (tr)

**Amaç:**

Relaps/refrakter Hodgkin lenfomanın (HL) otolog hematopoetik kök hücre nakli (OHKHN) sonrasındaki prognozunu gösterecek belirteçler ve çocukluk çağında yeni bir prognostik skorlama araştırıldı.

**Gereç ve Yöntemler:**

Bu çalışmada, Ocak 1990-Aralık 2014 tarihleri arasında OHKHN uygulanan 61 hastanın OHKHN sonrası prognozunu etkileyen faktörlerin sağkalım üzerine etkisi araştırıldı. Aynı zamanda Yaşa Göre Düzeltilmiş Uluslararası Prognostik İndeks ve Çocukluk Dönemi Uluslararası Prognostik İndeks’lerinin (ÇDUPİ) prognoz üzerindeki etkisi değerlendirildi.

**Bulgular:**

Altmış bir hastanın ortanca yaşı OHKHN sırasında 14,8 yıl (5-20 yıl) idi. Hastalardan, 28 olguda bir relaps, 8 olguda ≥2 relaps ve 25 olguda refrakter hastalık vardı. Kemosensitivite/kemoterapiye dirençlilik oranı 36/25 idi. Nakil öncesi radyoterapi almamak, nakil öncesi remisyonda olmamak, nakil sonrası beyaz kan hücresi sayısının 10x10^3^/µL üzerinde olması, nakil sonrası 100. gün pozitron emisyon tomografisi pozitifliği, tanıda 2,5 g/dL’den düşük serum albümin düzeyi progresyonsuz sağkalım üzerinde etkili belirteçler iken transplantasyon zamanı remisyonda olmamak, tanıda kemik iliği pozitifliği ve OHKHN sonrası relaps ise genel sağkalım üzerinde etkili parametreler olarak bulundu.

**Sonuç:**

Relaps/refrakter HL’li çocuklarda progresyonsuz ve genel sağkalımı etkileyen faktörler açıkça gösterildi. Serum laktat dehidrogenaz üst sınırını 500 IU/L olarak kullanan ÇDUPİ prognozu göstermede etkili bulundu. Relaps hastalarda ilk tam remisyonda OHKHN yapılmasını ve tanı anında yüksek prognostik skoru olan olguların da OHKHN açısından değerlendirmeye alınmasını önermekteyiz.

## INTRODUCTION

Children with Hodgkin’s lymphoma (HL) have an excellent prognosis, and their survival rates are satisfactory. The survival rates for HL have increased from 81% to 95% for children and adolescents, even those with advanced-stage disease [1]. Approximately 15% of patients cannot be cured and experience relapse after first-line treatment [2]. The relapse rate is increased up to 30% in advanced stages, and most relapses of HL occur within the first 2 years after completing treatment [3,4]. Autologous hematopoietic stem cell transplantation (AHSCT) is recommended for patients with refractory disease during therapy or disease relapse within 1 year after completing therapy [5,6]. However, some patients who undergo AHSCT develop recurrence within 1 year [7].

There has been much research on the prognostic parameters of patients who get worse [8,9,10,11,12]. These prognostic parameters and scores include the International Prognostic Index (IPI) and the Age-Adjusted IPI (AAIPI), which have been proven to show a close relationship between relapse and a poor prognosis [13]. However, most of these parameters and scores are for newly diagnosed and adult patients. Thus, in this multicenter study, we evaluated the effect of various factors and prognostic indexes, at diagnosis and pre- and posttransplant, on the relapse rate and survival of children with HL who undergo AHSCT. In addition, we aimed to create and develop a new international prognostic index that is specific to children with newly diagnosed advanced Hodgkin’s disease in order to evaluate the prognosis even after AHSCT.

## MATERIALS AND METHODS

This was a multicenter and retrospective study. Eleven pediatric stem cell transplant centers in different cities around Turkey participated, and the data were recorded retrospectively for 61 patients between January 1990 and December 2014. Three patients had undergone AHSCT before the year 2000, while 58 had undergone AHSCT after that point. Approval for the study was obtained from the local ethics committee. Exclusion criteria for the subjects included lymphomas other than HL and missing data. In four cases of an unknown pathologic subgroup of HL, the histologic slides were re-reviewed.

### Participants’ Characteristics

Sixty-one children from 11 centers, who underwent AHSCT for refractory or recurrent HL, were included and retrospectively analyzed. They showed some significant index values at diagnosis, pretransplant, and posttransplant for survival and event status after AHSCT.

Age, sex, concomitant diseases such as immune deficiency syndromes, and risk factors were analyzed. At diagnosis, risk factors were based on age, sex, primary tumor localization, number of relapses, time to relapse from diagnosis until completing the treatment, response to salvage chemotherapy, stage of disease, Karnofsky/Lansky score, pathologic HL type, bulky tumor, spleen involvement, extranodal involvement, bone or bone marrow involvement, B symptoms, hemoglobin, white blood cell (WBC) count, lymphocytes, monocytes, mean platelet volume (MPV), ferritin, albumin, lactate dehydrogenase (LDH), and sedimentation rate. Pretransplant risk factors were based on positron emission tomography-computed tomography (PET-CT) positivity, number of chemotherapy regimens, radiotherapy (RT), conditioning regimen, and disease status at the time of transplantation. After transplant, the risk factors were based on lymphocyte counts on posttransplant days 15 and 100, WBC counts, neutrophils, monocytes, platelets, MPV, neutrophil/lymphocyte ratio (NLR), platelet/lymphocyte ratio (PLR), LDH, and positivity of PET-CT on posttransplant day 100.

### Description of Features

**Chemosensitivity:** Susceptibility to the action of the chemotherapy protocol with a complete or very good response.

**Staging:** HL was graded using the Cotswold modification of the Ann Arbor staging system. Bulky disease is defined as the largest deposit being >10 cm or the mediastinum being wider than one-third of the chest on chest X-ray [14].

**Time to relapse:** Refractory disease was defined as occurring within 3 months after completion of therapy or during therapy. Early relapse was defined as disease recurring within 3-12 months, and late relapse was defined as disease occurring more than 12 months from the end of therapy [5].

### Prognostic Indexes

### Karnofsky/Lansky a Status

The Karnofsky/Lansky performance status is used to determine the functional status of the patient and is essential for all outcome-based analyses. The Karnofsky scale is designed for patients aged 16 years and older, and the Lansky scale is designed for those under 16 years. The Karnofsky/Lansky scores range from 100 to 0, with 100 indicating ‘perfect’ health and 0 representing death [15].

### Childhood International Prognostic Index

The original AAIPI incorporates serum LDH levels, Eastern Cooperative Oncology Group (ECOG) performance status, and Ann Arbor clinical stage at diagnosis. Based on these factors, patients are divided into four risk groups: 0, low risk; 1, low-intermediate risk; 2, high-intermediate risk; and 3, high risk [13]. We adapted the AAIPI for children according to an LDH level of 500 IU/L instead of 250 IU/L because of its high prognostic predictively in childhood HL, and we used the Cotswold modification of the Ann Arbor clinical stage at diagnosis with an ECOG modification score according to the pretransplant Karnofsky/Lansky performance score [15] and the Childhood International Prognostic Index (CIPI).

### Types of Outcome Measures

The definitions used as survival terms were as follows: 1) overall survival (OS) was calculated from the start of the treatment until death from any cause; 2) progression-free survival (PFS) was the achievement of stable disease without signs of progression, calculated from the day of transplant to the date of the next relapse or from the date of randomization for post-complete remission (CR) questions; and 3) event-free survival (EFS) was calculated from the date of the start of treatment to the date of the first event (failure to achieve CR, relapse, or death from any cause).

### Statistical Analysis

Statistical analyses were performed using SPSS 15.0. Descriptive analyses were presented using medians or mean ± standard deviation for variables. The Kaplan-Meier method and log-rank tests were used in the analysis. The risk factors described above were analyzed as prognostic factors for the survival rate with Cox regression analysis. Variables with values of p<0.05 were shown in the univariate analysis and were included in the multivariate analysis model. A p-value of less than 0.05 was considered to show a statistically significant result.

## RESULTS

### Clinical Features of Patients

Sixty-one patients with refractory/relapsed HL were analyzed. The demographic features are presented in Table 1. The median age was 14.8 years (minimum-maximum: 5-20), and the male/female ratio was 40/21=1.9. The subtypes of HL were lymphocyte-rich in three cases, nodular sclerosis in 32, mixed cellularity in 23, and unclassified in three. The chemosensitivity/chemorefractory ratio was 36/25. Additional conditions, such as tuberculosis (n=3), hepatitis B, Castleman disease, chronic persistent hepatitis, pericardial effusion, and thymoma, were detected in eight (13.1%) cases. The primary tumor localizations were cervical in 24 (39.3%) cases, mediastinal in 24 (39.3%), abdominal in nine (14.8%), inguinal in three (4.9%), and bone in one (1.6%). There was bulky tumor in 21 (34.4%) cases, extranodal involvement in 27 (44.3%), spleen involvement in 33 (54.1%), bone involvement in 12 (19.7%), and bone marrow involvement in one (1.6%). Stage I disease was detected in nine (14.7%) cases, Stage II in 12 (19.7%), Stage III in 10 (16.4%), and Stage IV in 30 (49.2%). Median (mean ± standard deviation, minimum-maximum) hemoglobin, WBC count, lymphocytes, monocytes, platelets, MPV, ferritin, albumin, LDH, erythrocyte sedimentation rate, and Karnofsky/Lansky score at diagnosis were 10.8 g/L (10.7±1.7 g/L, 5.9-13.1), 11.4x10^3^/µL (11.7±5.7x10^3^/µL, 3.7-24.2), 1.8x10^3^/µL (2±1.1x10^3^/µL, 0.5-4.7), 0.7x10^3^/µL (1.2±1.1x10^3^/µL, 0.2-6.4), 352x10^3^/µL (425-157x10^3^/µL, 193-797), 7.6 fL (7.5±1.1 fL, 5.3-9.7), 165 ng/dL (349±320 ng/dL, 20-1290), 3.7 mg/dL (3.8±0.8 mg/dL, 1.4-4.9), 419 IU/L (460±192 IU/L, 150-970), 63 mm/h (63±37 mm/h, 10-140), and 90 (88±16, 40-100), respectively.

Forty-two (68.8%) patients underwent RT before AHSCT. At the time of the study, 44 of 61 patients were alive, 15 were dead, and two were lost to follow-up. The causes of death were progressive disease in nine patients, infection in two patients, and other reasons except for relapse in four patients, including posttransplant lymphoproliferative disease in one case, acute myeloid leukemia progression in another, anaplastic large cell lymphoma as a secondary cancer in another, and transplant-related pulmonary hemorrhage in the last. The nonrelapse mortality rate was 40% (infection in two cases, other causes except for relapse in four cases, and lost to follow-up in two cases). The mortality rate 100 days after AHSCT was 6.5% (4 of 61 cases). The transplant-related mortality rate 100 days after AHSCT was 3.2% (2 of 61 cases; infection in one case and pulmonary hemorrhage in the other). The median relapse time was 11 months (minimum-maximum: 1-105). Types of relapse were refractory disease in 33 (54.1%) patients, early relapse in 12 (19.7%), and late relapse in 16 (26.2%). The PFS and OS rates for type of relapse were as follows: the 3-year PFS rates were 93.8% for late relapse, 91.7% for early relapse, and 38.7% for refractory disease (p<0.001); the OS rates were 100% for late relapse, 83.3% for early relapse (death due to other causes except for relapse and infection), and 57.6% for refractory disease (p=0.003). The 3-year survival rates were 57.6% for refractory disease and 88.9% for patients who responded (p=0.001). Three-year PFS rates were 38.7% for refractory disease and 94.4% for patients who responded (p<0.001).

### Features of Treatment

All of the patients underwent AHSCT. The disease status at the time of transplantation was as follows: 36 patients were in CR (CR2=28 patients, ≥CR3=8 patients) and 25 patients with refractory disease were not in remission. The 3-year PFS rates were 77.3% for patients with remission and 49.3% for patients without remission (p=0.007), while the OS rates were 91.4% for patients with remission and 59.1% for patients without remission (p=0.01).

The BEAM regimen was used in 44 (72.1%) patients, and other regimens were used in 17 (27.9%). Cyclophosphamide, etoposide, and BCNU in one case; busulphan and melphalan in five cases; busulphan, cyclophosphamide, and etoposide in four cases; fludarabine and busulphan in one case; total body irradiation, cyclophosphamide, and etoposide in three cases; CCNU, ifosfamide, and etoposide in two cases; and CCNU, cyclophosphamide, and etoposide in one case were used as other conditioning regimens. PET positivity and negativity were 37% and 63%, respectively, before AHSCT; however, these rates were 23% and 77% after AHSCT on day 100. Forty-two children (68.8%) received RT before AHSCT.

### Treatment Results

The median (mean ± standard deviation, minimum-maximum) level of lymphocyte counts at day 15 after AHSCT was 0.45x10^3^/µL (0.51±0.38x10^3^/µL, 0.01-1.8). The median (mean ± standard deviation, minimum-maximum) levels of WBCs, lymphocytes, neutrophils, monocytes, platelets, and MPV at day 100 after AHSCT were 3.9x10^3^/µL (4.6±2.7x10^3^/µL, 1.2-14.6), 1.3x103/µL (1.4±0.9x10^3^/µL, 0.02-5.1), 1.9x10^3^/µL (2.5±2.3x10^3^/µL, 0.3-12.4), 0.36x10^3^/µL (0.41±0.27x10^3^/µL, 0.04-1.43), 127x10^3^/µL (131±80x10^3^/µL, 14-308), and 7.5 fL (7.7±1.4 fL, 4.9-11.3), respectively. Three-year OS/PFS rates were 77.3% and 68.5% with a median follow-up of 27 months (minimum-maximum: 1-114 months) for all patients, respectively. The prognostic factors affecting EFS and OS are presented in Tables 2 and 3.

The 3-year PFS and OS rates were 65.2% and 78.4%, respectively, in patients who underwent AHSCT after the year 2000, but of the three cases recorded before the year 2000, two patients relapsed and all died.

**Figure 1:**
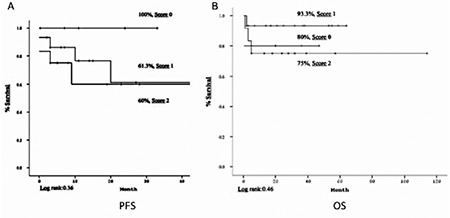
(A) Progression-free and (B) overall survival according to Childhood International Prognostic Index scores [254x135 mm (72x72 DPI)].

With regard to the CIPI scores, the 3-year PFS rates were 100% for a score of 0, 61.3% for a score of 1, and 60.0% for a score of 2 (p=0.36), while the 3-year OS rates were 80.0% for a score of 0 (death due to infection), 93.3% for a score of 1, and 75.0% for a score of 2 (p=0.46) (Figure 1).

Patients were scored based on a WBC count of >10x10^3^/µL at 100 days after AHSCT (0: no, 1: yes), RT before AHSCT (0: yes, 1: no), remission status at AHSCT (0: yes, 1: no), PET-CT status at 100 days after AHSCT (0: negative, 1: positive), and serum albumin of <2.5 g/dL (0: no, 1: yes). This scoring correlated with the following 3-year PFS rates: Group 0=100%, Group 1=66.7%, Group 2=50%, and Group >3=0% (p=0.001) (0: low risk, 1: low-intermediate risk, 2: high-intermediate risk, >3: high risk). For 3-year OS, remission status at AHSCT (0: yes, 1: no), relapse after AHSCT (0: no, 1: yes), and bone marrow positivity at diagnosis (0: no, 1: yes) were scored and showed that Group 0=92%, Group 1=78.6%, and Group 2=40% (p<0.001).

## DISCUSSION

Several prognostic factors affect survival in HL, such as extranodal disease at time of relapse, mediastinal mass at time of transplant, advanced stage at relapse, primary refractory disease, and a positive PET scan prior to AHSCT [2,16,17,18,19]. We found that no pretransplant RT, a posttransplant WBC count of >10x10^3^/µL, posttransplant PET positivity at day 100, serum albumin of <2.5 g/dL at diagnosis, no remission at the time of transplantation, bone marrow positivity at diagnosis, and relapse after AHSCT were significant parameters related to events after AHSCT and OS. In addition, CIPI was significant in estimating survival after AHSCT in HL.

RT is an important treatment method for HL. Although there have been several studies on discarding RT in the treatment of HL at earlier stages, its removal has been proven to increase the risk of relapse at later stages [20]. Some centers also tend to give RT after transplantation in order to not waste time before the transplant and to avoid recurrence of the disease. Our results clearly showed that there was a 13.2-fold increased risk of relapse in patients who underwent RT after transplant, but there was no impact on OS. Lowering the tumor volume with RT before AHSCT may improve the prognosis.

The NLR and PLR were reported to indicate disease severity and prognosis in patients with various diseases [21]. However, there is no relationship between pretransplant and posttransplant NLR, PLR, lymphocyte, monocyte, and MPV levels in patients who survive. There is also no relationship between pre- and posttransplant neutrophil, lymphocyte, NLR, PLR, and MPV levels and survival. However, on posttransplant day 100, a WBC count of over 10x10^3^/µL was found to increase the relapse risk 32.8-fold, but it had no impact on OS.

Several prognostic indexes have been used to evaluate the survival of patients with lymphoma. The IPI and the AAIPI are the two of the leading methods [13]. Low scores are correlated with a high survival rate and high scores with a low survival rate. Satwani et al. [22] reported that patients with relapsed/refractory HL in the high-risk group according to prognostic models for PFS have a high progression risk after AHSCT. We used the CIPI and it worked well for estimating survival. The survival rates of our patients were 100%, 59.3%, and 58.3% for events after AHSCT and 80%, 91.7%, and 80% for OS for scores of 0, 1, and 2, respectively. According to our study, the risk of progression increased with the increase of the prognostic score, and this result was compatible with previous studies.

Some pediatric oncology centers may prefer to treat their patients with chemotherapy without AHSCT. However, the outcomes of patients who underwent salvage chemotherapy alone were not found to be satisfactory [23]. Studies about the proper timing of AHSCT are limited in pediatric populations. Stoneham et al. reported that AHSCT did not offer any significant survival advantages over conventional salvage therapy in children with relapsed HL compared to those with primary refractory disease [24]. Ataş et al. did not demonstrate survival improvement after AHSCT in early-relapse HL cases (n=6) when compared to late-relapse cases (n=3). Therefore, AHSCT is advisable regardless of the time of relapse in children with relapsed HL [25]. In our study, relapse occurred in 61 subjects with HL; of these, 33 cases (54.1%) were reported to be refractory to treatment, 12 (19.7%) were early relapses, and 16 (26.2%) were late relapses. Early relapse and refractory disease were three times more common than late relapse, and the OS rates were 100% for late relapse, 83.3% for early relapse, and 57.6% for refractory disease at 3 years.

Metzger et al. reported treatment results after initial salvage therapy according to early relapse (28%), late relapse (42%), and refractory disease (30%). However, inadequate response to initial salvage therapy was the only significant variable with regard to prognosis, and the 5-year OS rate for these patients was 17.9%, compared with 97.2% for the patients who responded [2]. In our study, the 5-year OS rate for patients with chemorefractory disease was 40.3%, compared to 88.9% for patients who responded. Based on these studies, it seems that chemorefractory patients have poor survival rates even after undergoing AHSCT.

Disease status at transplantation (with or without CR) is one of the most important risk factors for outcomes. Marcais et al. reported a 39% OS rate and an 18% PFS rate for chemorefractory disease compared to patients in CR, who had a 70%-74% OS rate and a 40%-51% PFS rate at 3 years [26]. In our study, 36 patients were in CR and 25 patients were without remission. The survival rates of patients in remission prior to transplant were two times higher than those of patients without remission (OS: 91.4% vs. 59.1%). The relapse rate was lower in the remission group (PFS: 77.3% vs. 49.3%). If the tumor burden can be lowered sufficiently, the success of AHSCT treatment may increase, with high survival and low relapse rates.

The benefit of stem cell transplantation was mainly seen in PFS for patients with relapsed/refractory HL after first-line therapy [27]. Some authors recommended AHSCT for children with early relapsed and refractory HL [5,6,28]. The 3-year OS rate for patients who underwent AHSCT was 77.3% in this study, and their PFS rate was 68.5%. Other studies on AHSCT reported the projected survival rate as 45% to 70% and PFS as 30% to 89% [16,17,18,29]. Despite our short median follow-up period (27 months), our results are comparable to those found in other reports of children with relapsed/refractory HL who received AHSCT.

Pretreatment factors such as advanced stage of disease (Stage IIB, IIIB, or IV), presence of B symptoms, histology, presence of bulky disease, extranodal extension, elevated erythrocyte sedimentation rate, leukocytosis (WBC count of ≥11.5x10^3^/µL), anemia (hemoglobin of <10.5-11.0 g/L), male sex, rapidity of response to initial treatment with chemotherapy, fluorodeoxyglucose-PET avidity after two cycles, low serum albumin (<4 g/dL), and low lymphocyte count (<0.6x10^3^/µL or <8% of WBC count) were reported as prognostic factors in previous studies [8,9,10,11,12,30]. Relapsed patients with HL who had localized disease that was treated with chemotherapy alone and/or low-dose involved-field radiation therapy consolidation, and whose relapse occurred ≥12 months after completing therapy, have better survival with intensive conventional chemotherapy [8]. Extranodal disease at relapse, mediastinal mass at time of transplant, advanced stage at relapse, primary refractory disease, and a positive PET scan prior to AHSCT were significant factors in post-AHSCT events [2,16,17,18,19]. Claviez et al. reported that the most important predictors for disease control following AHSCT were time to relapse and disease status at transplantation [5]. We found by multivariate analysis that a WBC count of >10x10^3^/µL at 100 days after AHSCT, no RT before AHSCT, no remission after AHSCT, PET-CT positivity at 100 days after AHSCT, and serum albumin of <2.5 g/dL were significant factors for PFS, and that no remission after AHSCT, relapse after AHSCT, and bone marrow positivity at diagnosis were significant factors for OS.

In conclusion, the major factors affecting the prognosis of children with relapsed/refractory HL seem to be tumor load and chemosensitivity. Treatments that significantly decrease the tumor volume before AHSCT may improve the survival rate, as we saw a benefit with RT on EFS when performed before AHSCT. In addition, the survival rates of patients in remission before AHSCT were twice as high as those of patients without remission. AHSCT had a significant benefit on OS, but the timing must be investigated in larger studies. We also suggest different treatment approaches for patients with high IPI and/or CIPI scores to improve EFS and PFS. We suggest that a CIPI that uses an LDH level of 500 IU/L is more useful in childhood. A serum albumin status of <2.5 g/dL at diagnosis had a significant effect on PFS, pointing to the study of the immunologic profile of the patients, and the treatment schedule may be redesigned with this immunologic profile. In addition, the characteristics that showed significance in a univariate but not a multivariate analysis appear to have an influence as well, and might show a stronger correlation in larger trials. Patients with high prognostic factors should be evaluated at diagnosis and may be directed to AHSCT consolidation therapy at the time of the first CR.

## Ethics

Ethics Committee Approval: Gülhane Military Medical Academy Ethic Committee 03.02.2015/03, Informed Consent: Retrospective study.

## Figures and Tables

**Table 1:**
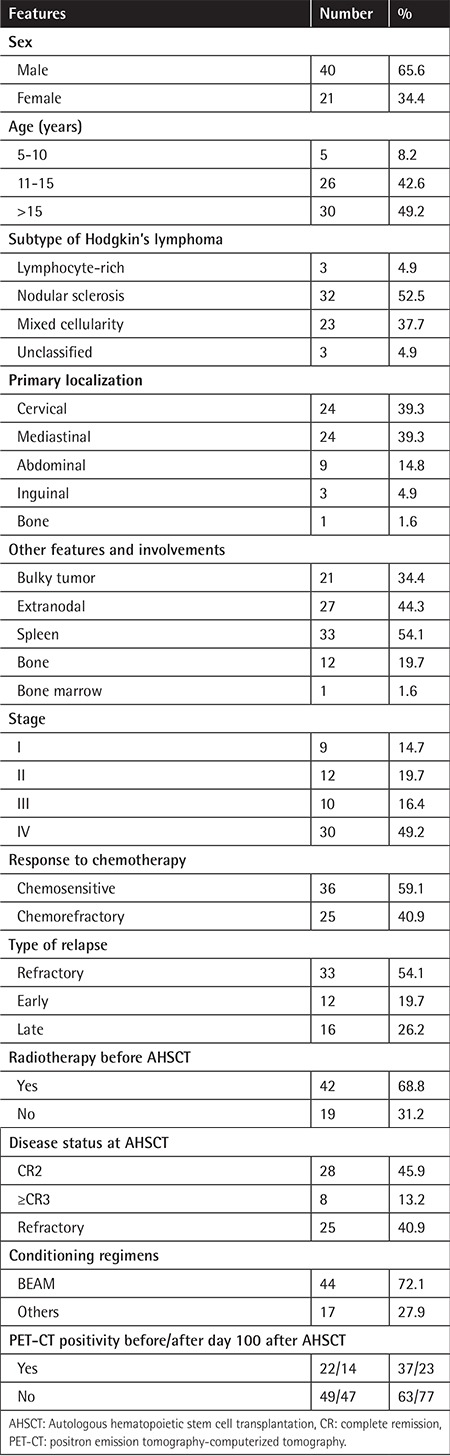
Demographic, clinical, and histopathological features of the patients with relapsed refractory Hodgkin’s lymphoma.

**Table 2:**
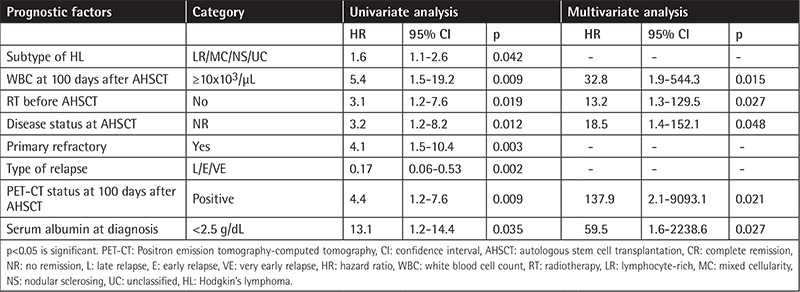
Effective parameters on relapse of patients with Hodgkin’s lymphoma after autologous hematopoietic stem cell transplantation in univariate and multivariate analysis.

**Table 3:**
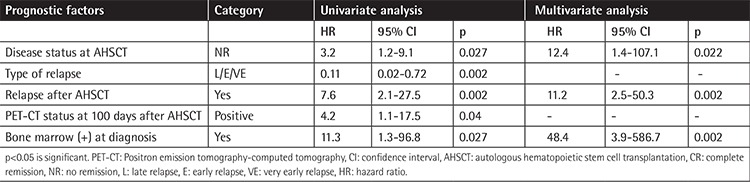
Effective parameters on survival of patients with Hodgkin’s lymphoma after AHSCT in univariate and multivariate analysis.

## References

[1] Smith MA, Seibel NL, Altekruse SF, Ries LA, Melbert DL, O’Leary M, Smith FO, Reaman GH (2010). Outcomes for children and adolescents with cancer: challenges for the twenty-first century. J Clin Oncol.

[2] Metzger ML, Hudson MM, Krasin MJ, Wu J, Kaste SC, Kun LE, Sandlund JT, Howard SC (2010). Initial response to salvage therapy determines prognosis in relapsed pediatric Hodgkin lymphoma patients. Cancer.

[3] Voss SD, Chen L, Constine LS, Chauvenet A, Fitzgerald TJ, Kaste SC, Slovis T, Schwartz CL (2012). Surveillance computed tomography imaging and detection of relapse in intermediate- and advanced-stage pediatric Hodgkin’s lymphoma: a report from the Children’s Oncology Group. J Clin Oncol.

[4] Kuruvilla J (2009). Standard therapy of advanced Hodgkin lymphoma. Hematology Am Soc Hematol Educ Program.

[5] Claviez A, Sureda A, Schmitz N (2008). Haematopoietic SCT for children and adolescents with relapsed and refractory Hodgkin’s lymphoma. Bone Marrow Transplant.

[6] Körholz D, Claviez A, Hasenclever D, Kluge R, Hirsch W, Kamprad F, Dörffel W, Wickmann L, Papsdorf K, Dieckmann K, Kahn T, Mauz-Körholz C, Dannenberg C, Pötter R, Brosteanu O, Schellong G, Sabri O (2004). The concept of the GPOH-HD 2003 therapy study for pediatric Hodgkin’s disease: evolution in the tradition of the DAL/GPOH studies. Klin Padiatr.

[7] Dhakal S, Biswas T, Liesveld JL, Friedberg JW, Phillips GL, Constine LS (2009). Patterns and timing of initial relapse in patients subsequently undergoing transplantation for Hodgkin’s lymphoma. Int J Radiat Oncol Biol Phys.

[8] Nachman JB, Sposto R, Herzog P, Gilchrist GS, Wolden SL, Thomson J, Kadin ME, Pattengale P, Davis PC, Hutchinson RJ, White K, Children’s Cancer Group (2002). Randomized comparison of low-dose involved-field radiotherapy and no radiotherapy for children with Hodgkin’s disease who achieve a complete response to chemotherapy. J Clin Oncol.

[9] Rühl U, Albrecht M, Dieckmann K, Lüders H, Marciniak H, Schellenberg D, Wickmann L, Dörffel W (2001). Response-adapted radiotherapy in the treatment of pediatric Hodgkin’s disease: an interim report at 5 years of the German GPOH-HD 95 trial. Int J Radiat Oncol Biol Phys.

[10] Smith RS, Chen Q, Hudson MM, Link MP, Kun L, Weinstein H, Billett A, Marcus KJ, Tarbell NJ, Donaldson SS (2003). Prognostic factors for children with Hodgkin’s disease treated with combined-modality therapy. J Clin Oncol.

[11] Carde P, Koscielny S, Franklin J, Axdorph U, Raemaekers J, Diehl V, Aleman B, Brosteanu O, Hasenclever D, Oberlin O, Bonvin N, Björkholm M (2002). Early response to chemotherapy: a surrogate for final outcome of Hodgkin’s disease patients that should influence initial treatment length and intensity?. Ann Oncol.

[12] Metzger ML, Castellino SM, Hudson MM, Rai SN, Kaste SC, Krasin MJ, Kun LE, Pui CH, Howard SC (2008). Effect of race on the outcome of pediatric patients with Hodgkin’s lymphoma. J Clin Oncology.

[13] No authors listed (1993). A predictive model for aggressive non-Hodgkin’s lymphoma. The International Non-Hodgkin’s Lymphoma Prognostic Factors Project. N Engl J Med.

[14] Lister TA, Crowther D, Sutcliffe SB, Glatstein E, Canellos GP, Young RC, Rosenberg SA, Coltman CA, Tubiana M (1989). Report of a committee convened to discuss the evaluation and staging of patients with Hodgkin’s disease: Cotswolds meeting. J Clin Oncol.

[15] (2009). National Marrow Donor Program & The Medical College of Wisconsin. Forms Manual: Appendix-L-Karnofsky/Lansky Performance Status A00428 Revision 1.

[16] Lieskovsky YE, Donaldson SS, Torres MA, Wong RM, Amylon MD, Link MP, Agarwal R (2004). High-dose therapy and autologous hematopoietic stem-cell transplantation for recurrent or refractory pediatric Hodgkin’s disease: results and prognostic indices. J Clin Oncol.

[17] Akhtar S, Abdelsalam M, El Weshi A, Al Husseini H, Janabi I, Rahal M, Maghfoor I (2008). High-dose chemotherapy and autologous stem cell transplantation for Hodgkin’s lymphoma in the kingdom of Saudi Arabia: King Faisal specialist hospital and research center experience. Bone Marrow Transplant.

[18] Harris RE, Termuhlen AM, Smith LM, Lynch J, Henry MM, Perkins SL, Gross TG, Warkentin P, Vlachos A, Harrison L, Cairo MS (2011). Autologous peripheral blood stem cell transplantation in children with refractory or relapsed lymphoma: results of Children’s Oncology Group study A5962. Biol Blood Marrow Transplant.

[19] Jabbour E, Hosing C, Ayers G, Nunez R, Anderlini P, Pro B, Khouri I, Younes A, Hagemeister F, Kwak L, Fayad L (2007). Pretransplant positive positron emission tomography/gallium scans predict poor outcome in patients with recurrent/refractory Hodgkin lymphoma. Cancer.

[20] Wolden SL, Chen L, Kelly KM, Herzog P, Gilchrist GS, Thomson J, Sposto R, Kadin ME, Hutchinson RJ, Nachman J (2012). Long-term results of CCG 5942: a randomized comparison of chemotherapy with and without radiotherapy for children with Hodgkin’s lymphoma--a report from the Children’s Oncology Group. J Clin Oncol.

[21] Azab B, Shah N, Radbel J, Tan P, Bhatt V, Vonfrolio S, Habeshy A, Picon A, Bloom S (2013). Pretreatment neutrophil/lymphocyte ratio is superior to platelet/lymphocyte ratio as a predictor of long-term mortality in breast cancer patients. Med Oncol.

[22] Satwani P, Ahn KW, Carreras J, Abdel-Azim H, Cairo MS, Cashen A, Chen AI, Cohen JB, Costa LJ, Dandoy C, Fenske TS, Freytes CO, Ganguly S, Gale RP, Ghosh N, Hertzberg MS, Hayashi RJ, Kamble RT, Kanate AS, Keating A, Kharfan-Dabaja MA, Lazarus HM, Marks DI, Nishihori T, Olsson RF, Prestidge TD, Rolon JM, Savani BN, Vose JM, Wood WA, Inwards DJ, Bachanova V, Smith SM, Maloney DG, Sureda A, Hamadani M (2015). A prognostic model predicting autologous transplantation outcomes in children, adolescents and young adults with Hodgkin lymphoma. Bone Marrow Transplant.

[23] Longo DL, Duffey PL, Young RC, Hubbard SM, Ihde DC, Glatstein E, Phares JC, Jaffe ES, Urba WJ, DeVita VT (1992). Conventional-dose salvage combination chemotherapy in patients relapsing with Hodgkin’s disease after combination chemotherapy: the low probability for cure. J Clin Oncol.

[24] Stoneham S, Ashley S, Pinkerton CR, Wallace WH, Shankar AG, United Kingdom Children’s Cancer Study Group (2004). Outcome after autologous hemopoietic stem cell transplantation in relapsed or refractory childhood Hodgkin disease. J Pediatr Hematol Oncol.

[25] Ataş E, Kesik V, Babacan O, Korkmazer N, Akyüz C (2015). The timing of autologous stem cell transplantation and the prognostic factors affecting the prognosis in children with relapsed Hodgkin lymphoma. Pediatr Transplant.

[26] Marcais A, Porcher R, Robin M, Mohty M, Michalet M, Blaise D, Tabrizi R, Clement L, Ceballos P, Daguindau E, Bilger K, Dhedin N, Lapusan S, Bay JO, Pautas C, Garban F, Ifrah N, Guillerm G, Contentin N, Bourhis JH, Yakoub Agha I, Bernard M, Cornillon J, Milpied N (2013). Impact of disease status and stem cell source on the results of reduced intensity conditioning transplant for Hodgkin’s lymphoma: a retrospective study from the French Society of Bone Marrow Transplantation and Cellular Therapy (SFGM-TC). Haematologica.

[27] Rancea M, Monsef I, von Tresckow B, Engert A, Skoetz N (2013). High-dose chemotherapy followed by autologous stem cell transplantation for patients with relapsed/refractory Hodgkin lymphoma. Cochrane Database Syst Rev.

[28] Schellong G, Dörffel W, Claviez A, Körholz D, Mann G, Scheel-Walter HG, Bökkerink JP, Riepenhausen M, Lüders H, Pötter R, Rühl U, DAL/GPOH (2005). Salvage therapy of progressive and recurrent Hodgkin’s disease: results from a multicenter study of the pediatric DAL/GPOH-HD study group. J Clin Oncol.

[29] Shafer JA, Heslop HE, Brenner MK, Carrum G, Wu MF, Liu H, Ahmed N, Gottschalk S, Kamble R, Leung KS, Myers GD, Bollard CM, Krance RA (2010). Outcome of hematopoietic stem cell transplant as salvage therapy for Hodgkin’s lymphoma in adolescents and young adults at a single institution. Leuk Lymphoma.

[30] Hasenclever D, Diehl V (1998). A prognostic score for advanced Hodgkin’s disease. International Prognostic Factors Project on Advanced Hodgkin’s Disease. N Engl J Med.

